# Improvement of platelet dysfunction in chronic myelogenous leukemia following treatment with imatinib: a case report

**DOI:** 10.1186/1752-1947-5-215

**Published:** 2011-05-30

**Authors:** Alexander Shimabukuro-Vornhagen, Achim Rothe, Lucia Nogova, Matthias Kochanek, Christoph Scheid, Michael von Bergwelt-Baildon

**Affiliations:** 1 Department I of Internal Medicine, University Hospital Cologne, Kerpener Strasse 62, D-50924 Köln, Germany

## Abstract

**Introduction:**

In patients with chronic myeloid leukemia, tyrosine kinase inhibitors suppress the *BCR-ABL*^+ ^clone and often induce complete molecular remissions. Megakaryocytes in such patients have been shown to be derived from the *BCR-ABL*^+ ^clone, and abnormal platelet function is frequent in chronic myeloid leukemia. However, little is known about the influence of modern targeted therapy on chronic myeloid leukemia-associated platelet disorders.

**Case presentation:**

We report the case of a massive hemorrhage in a 32-year-old Caucasian man caused by chronic myeloid leukemia-associated platelet dysfunction, which improved after treatment with imatinib.

**Conclusion:**

This report demonstrates that platelet dysfunction and bleeding disorder in *BCR-ABL*^+ ^chronic myeloid leukemia can successfully be treated with imatinib. We suggest the monitoring of platelet function in future studies using imatinib to treat patients with chronic myeloid leukemia.

## Introduction

Chronic myeloid leukemia (CML) is a clonal myeloproliferative disorder that results from the malignant transformation of a hematopoietic stem cell. It is characterized by the Philadelphia chromosome (Ph), which is formed by translocation and fusion of the long arms of chromosomes 9 and 22 in a pluripotent hematopoietic progenitor cell. At the molecular level, the fusion generates a BCR-ABL protein with constitutive tyrosine kinase activity. Tyrosine kinase inhibitors such as imatinib are able to suppress the *BCR-ABL*^+ ^clone and induce molecular remission. Megakaryocytes are thought to belong to the *BCR-ABL*^+ ^clone, and abnormal platelet function has been described in CML. However, little is known about the influence of modern targeted therapy on CML-associated platelet disorders. We report the case of a massive hemorrhage caused by CML-associated platelet dysfunction which improved after treatment with imatinib.

## Case presentation

A 32-year-old Caucasian man consulted his primary care physician because of left upper-quadrant abdominal pain, dyspnea upon exertion, drenching night sweats, and unintentional weight loss. His initial clinical examination revealed tachycardia of 120 beats/minute, pronounced pallor, and splenomegaly of 8 cm×26 cm. Laboratory analysis showed a leukocyte count of 327×10^9^/L, a hemoglobin level of 7.0 g/dL, and a platelet count of 377×10^9^/μL. The patient's lactate dehydrogenase level was significantly elevated at 1080 IU/L. His coagulation tests showed a normal activated partial thromboplastin time (aPTT) of 34 seconds (< 36 seconds) and an international normalized ratio (INR) of 1.1. The patient was taking no medications.

He was immediately referred to our Department of Hematology and Oncology. There a bone marrow aspiration was performed, and cytomorphologic as well as cytogenetic analysis was carried out. Microscopic examination showed a left-shifted granulopoiesis with 8% blasts and 20% promyelocytes. The alkaline phosphatase index was 2. A *BCR-ABL *fusion transcript (coefficient 26.074 = 100×bcr-abl/abl) could be identified by polymerase chain reaction assay. Thus, the diagnosis of a Ph^+ ^CML in chronic phase was made.

The patient initially underwent leukapheresis twice and then received cytoreductive chemotherapy with hydroxyurea. Simultaneously, the search for a bone marrow donor was initiated. After 14 days, normal leukocyte counts were reached and the therapeutic regimen was switched to the tyrosine kinase inhibitor imatinib. While the initial management efficiently led to a reduction of CML blasts, the patient encountered a severe complication of a standard diagnostic procedure: three days after bone marrow aspiration at the left superior posterior iliac spine he complained about massive pain in his left buttock. A clinical examination revealed massive swelling that was extremely tender to touch. The increasing pain necessitated the use of continuous intravenous morphine. Computed tomography revealed a large hematoma in the left gluteal region. One day after the onset of symptoms surgery became necessary because of increasing swelling accompanied by a drop of the hemoglobin and impending compartment syndrome. During surgery, diffuse bleeding was noted without an identifiable lesion to major vessels or other anatomic structures. The hematoma (about 500 mL) was evacuated, and fresh frozen plasma (FFP) as well as packed red blood cells (PRBCs) were given to the patient. At the time of surgery, PTT was 40 seconds, the INR was 1.4, and the platelet count was 300×10^9^/L. The patient's post-operative course was complicated by continuous bleeding with a further increase in size of the hematoma and extension into the left leg, renewed deterioration of coagulation parameters, and onset of fever (< 38.5°C). Continuous substitution of FFP and PRBCs was necessary, and surgery had to be repeated on day 9. Because of continuous post-operative bleeding and the risk of compartment syndrome, the wound was left open to reduce tissue pressure. The bleeding time assessed according to the modified template method was prolonged at 13 minutes. Therefore, further platelet function tests were performed. Occlusion times determined by using a platelet function analyzer (PFA-100; Dade Behring, Inc., Deerfield, IL, USA) were 152 seconds for adenosine diphosphate (ADP) (71 to 118 seconds) and > 300 seconds for collagen/epinephrine (C/Epi) (94 to 193 seconds), respectively. As the patient had no history of bleeding prior to the diagnosis of CML, further laboratory examinations for other pathological bleeding situations were not performed. Therefore, despite increased platelet counts, platelet transfusions were initiated. Following the platelet substitution, his bleeding ceased quickly. The patient could be discharged 10 days later. At that time, the patient had paresis of the leg with a positive Trendelenburg's sign due to compression of the superior gluteal nerve. His bleeding time was still highly pathologic upon discharge.

We assumed that the patient's platelet dysfunction originated from a clonal expansion of dysfunctional megakaryocytes derived from the identical stem cell from which the CML blasts had originated. We therefore hypothesized that treatment targeting BCR-ABL would be equally effective in reducing the CML blasts and dysfunctional megakaryocytes. Despite the risk of hemorrhage in this patient, we therefore continued a single-agent treatment strategy with imatinib in an outpatient setting.

No further episodes of bleeding occurred during the follow-up of the patient. During that time, the patient underwent dental surgery without experiencing excessive bleeding. The defect in the gluteal region disappeared completely as a result of secondary wound healing, and the paresis regressed steadily. Six months after discharge from the hospital the patient was in excellent health. At his last complete laboratory follow-up four years later, his bleeding time was normal (four minutes) and his C/Epi occlusion time was 211 (94 to 193 seconds). He was in complete molecular remission. Six years after the incident the patient remains clinically in very good health without any signs of bleeding and continues to be in complete molecular remission.

## Discussion

The pathologic changes in CML affect not only CML blasts but also other hematopoietic lineages, which adds to the array of clinical manifestations and increases morbidity as well as mortality of the disease [1-4]. Bleeding is a frequent complication in patients with myeloproliferative disorders, including CML, and qualitative platelet abnormalities are frequently observed [5]. Thrombocytosis is observed in about one of three patients, and platelet dysfunction occurs in one of six patients. Platelet dysfunction in chronic myeloproliferative disorders is multi-factorial [6]. One of the most characteristic platelet defects in myeloproliferative disorders is a reduced responsiveness to epinephrine, while the response to ADP usually is intact. This was found to be due to a reduction in α-adrenergic receptors on the platelet membrane. The cause of the decreased surface expression of α-adrenergic receptors is unknown, though.

Molecularly, *BCR-ABL *rearrangements have clearly been demonstrated in megakaryocytic cell lines as well as in megakaryocytes from the vast majority of CML patients [1,3,4,7]. Therapeutic approaches in Ph^+ ^CML to reduce the number of CML blasts previously had proven effective in reducing the number of *BCR-ABL*^+ ^megakaryocytes [3,7]. Furthermore, previous studies in which imatinib was used to treat patients with CML confirmed *in vitro *effects on other *BCR-ABL*^+ ^subpopulations. Importantly, bone marrow biopsies in these patients demonstrated an inhibitory effect of imatinib on increased megakaryopoeisis [8]. Also, Thiele *et al*. [9] found that the atypical micromegakaryocytes that are prevalent in Ph^+ ^CML were significantly reduced after imatinib therapy with a further reappearance of normal-sized megakaryocytes.

The study by Nolte *et al*. [10] showed that not only the transforming event but also the Ph^+ ^translocation leading to *BCR-ABL*^+ ^fusion happens prior to the differentiation of the pluripotent stem cell into different myeloid lineages.

*In vitro *studies done by Era [11] and Buet *et al*. [12] showed two novel aspects of the differentiation of hematopoietic stem cells. Era demonstrated that enforced *BCR-ABL*^+ ^expression was sufficient to increase the number of both multi-lineage progenitors and myeloid progenitors. Buet *et al*. showed that alteration of signal transduction via *BCR-ABL*^+ ^reprograms megakaryocyte cells into erythroid cells by down-regulation of FLI-1. This suggests that *BCR-ABL*^+ ^represents a "molecular switch" for the decision for growth and differentiation in hematopoietic stem cells in general [12].

**Figure 1:**
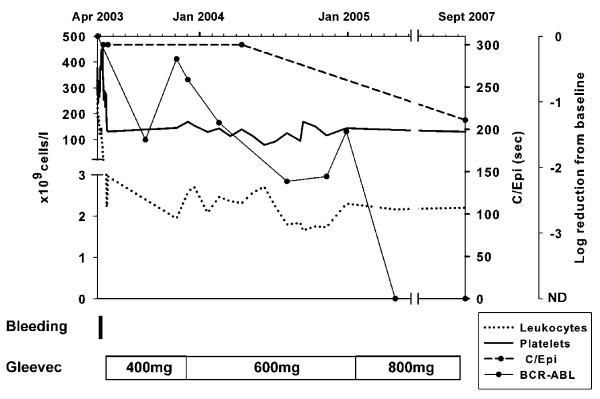
**Time course of platelet counts, leukocyte counts, quantitative polymerase chain reaction for BCR-ABL (expressed as log reduction from baseline), and platelet function analyzer PFA-100 platelet function tests**.

On the basis of the high frequency of megakaryocyte involvement in CML patients and the concurrent thrombocytosis in our patient, we assumed that platelet dysfunction was a manifestation of CML and that the megakaryocytes also carried the *BCR-ABL *fusion gene. Interestingly, in our patient, the achievement of complete molecular remission was paralleled by a significant improvement in platelet dysfunction (Figure 1). This was reflected in normalization of bleeding time, a decrease in C/Epi from > 300 seconds to 211 seconds, and successful dental surgery without bleeding complications.

Because of the patient's clinical course and his reluctance, we decided not to perform another bone marrow biopsy to confirm *BCR-ABL *fusion transcripts in his megakaryocytes.

## Conclusion

To our knowledge, this is the first report to show that platelet dysfunction and bleeding disorder in *BCR-ABL*^+ ^CML can be treated successfully with imatinib. To validate these observations, we strongly suggest monitoring platelet function in future studies using imatinib or second-generation tyrosine kinase inhibitors, such as dasatinib, in patients with CML. We emphasize the need for further research to validate our clinical observations.

## Consent

Written informed consent was obtained from the patient for publication of this case report and any accompanying images. A copy of the written consent is available for review by the Editor-in-Chief of this journal.

## Competing interests

The authors declare that they have no competing interests.

## Authors' contributions

ASV wrote the case report. AR contributed to the writing of the case report. LN, MK, and CS analyzed and interpreted the patient data regarding the hematological disease. MvBB analyzed and interpreted the patient data regarding the hematological disease and contributed to the writing and revision of the manuscript. All authors read and approved the final manuscript.
